# Establishing Bedding Requirements during Transport and Monitoring Skin Temperature during Cold and Mild Seasons after Transport for Finishing Pigs

**DOI:** 10.3390/ani4020241

**Published:** 2014-05-21

**Authors:** John McGlone, Anna Johnson, Avi Sapkota, Rebecca Kephart

**Affiliations:** 1 Department of Animal and Food Sciences, Texas Tech University, Lubbock, TX 79409, USA; E-Mail: asapkota@purdue.edu; 2 Department of Animal Science, Iowa State University, Ames, IA 50011, USA; E-Mails: johnsona@iastate.edu (A.J.); rkdavis@iastate.edu (R.K.)

**Keywords:** bedding, microenvironment, pigs, skin surface temperature, transportation, welfare

## Abstract

**Simple Summary:**

Typically, bedding is used to improve pig comfort and welfare during transport. This study assesses the level of bedding required during transport of finishing pigs in semi-truck trailers. The present study shows that adding more than six bales/trailer of bedding in cold weather and more than three bales/trailer of bedding in mild weather provides no benefit to the pigs. Economic forces would not favor increased bedding with no benefit. Use of infrared thermography may provide a useful tool to indicate when cooling interventions are needed during warm weather.

**Abstract:**

The broad aim of this study was to determine whether bedding level in the transport trailer influenced pig performance and welfare. Specifically, the objective was to define the bedding requirements of pigs during transportation in commercial settings during cold and mild weather. Animals (n = 112,078 pigs on 572 trailers) used were raised in commercial finishing sites and transported in trailers to commercial processing plants. Dead on arrival (DOA), non-ambulatory (NA), and total dead and down (D&D) data were collected and skin surface temperatures of the pigs were measured by infrared thermography. Data were collected during winter (Experiment 1) and fall/spring (Experiment 2). Total D&D percent showed no interaction between bedding level and outside air temperature in any experiments. Average skin surface temperature during unloading increased with outside air temperature linearly in both experiments (*P* < 0.01). In conclusion, over-use of bedding may be economically inefficient. Pig skin surface temperature could be a useful measure of pig welfare during or after transport.

## 1. Introduction

More than 100 million pigs are transported to processing plants yearly in the U. S. [1]. Losses from dead and down (D&D) pigs arriving at the processing plants represent a significant financial loss [2] and suggest an animal welfare concern. Losses have been defined as any mortality or non-ambulatory pigs occurring during transportation from finishing sites to the processing plant. These losses include:

- Dead on arrival (DOA) defined as pigs that die during transit; and
- Non-ambulatory (NA), which are pigs unable to move or keep up with other unloaded pigs and have to be killed in suspect of disease. The NA category can be delineated into two further sub-categories:
  ○ Non-ambulatory, non-injured (NANI) defined as pigs that cannot move or walk, but do not have any obvious signs of injury, trauma, or disease; and
  ○ Non-ambulatory, injured (NAI), pigs that cannot move or walk due to injury during any stage of transportation and show clear signs of injury, trauma, or disease [2].

Factors that may influence losses during transit include: micro-environment inside the trailer, stocking density, animal health, transit time, wait time both at the farm (from loading to leaving) and plant (prior to unloading) [2,3], and handling of the pigs throughout the entire process [4]. Temperature, humidity, air speed, air pressure, level of gases (ammonia, carbon dioxide), and the amount and condition of any bedding have been identified as major microenvironment components that may affect transport losses [5,6]. The level of bedding material may be a crucial component in controlling the microenvironment and thereby improve swine welfare during transport.

The National Pork Board introduced a training program for swine transporters, producers, and handlers, now known as the Transport Quality Assurance (TQA) program, in 2002. As new research is done in swine transport, the National Pork Board updates the TQA, with the most recent edition published in 2008 [7]. This program outlines recommendations to help those in the pork industry understand handling and transport of pigs. The TQA Handbook gives the following guidelines regarding bedding: at temperatures lower than −12 °C, pigs should be provided with four bags of bedding (heavy); at temperatures of −12 °C to 3.8 °C, pigs should be provided with three bags of bedding (medium); and at temperatures above 3.8 °C, pigs should be provided with two bags of bedding (light). These recommendations were based on professional judgment and experience as opposed to scientific data. Therefore, the present study was designed to determine the level of bedding that might decrease transport losses, thereby improving pig welfare and lowering economic losses.

## 2. Experimental Section

### 2.1. General

Institutional Animal Care and Use Committees at Texas Tech- and Iowa State University approved the experimental protocol for this study. This research was conducted using commercial finishing sites, trailers, and processing plants. A total of 112,078 finisher barrows and gilts (mixed commercial genetics) from farms in Iowa and Minnesota were observed during loading and unloading from trailers going to commercial processing plants in Missouri or Iowa. Trailers were primarily pot-belly styles (center portion lower than the top of the tires), though some straight-deck styles (each of 2 decks were level) were also used (1.7% of trailers were straight deck). Trailer dimensions were 14.7 m long × 2.6 m wide or 16.3 m long × 2.6 m wide. Each trailer transported an average of 166.9 ± 0.63 pigs (minimum = 27, maximum = 193). Duration of travel varied due to varying distances between finishing sites and processing plants. The processing plants that cooperated wish to remain anonymous.

### 2.2. Treatments

This study was divided into 2 experiments: cold and mild weather. Each bale of wood shavings used was 22.7 kg and 0.2 m^3^. Bedding levels were assigned randomly to trailers and applied by the 13 transport companies. A single bedding level was assigned to each trailer, with the same amount of bedding applied to each deck. Researchers confirmed the amount of bedding was appropriate. Weather data were available on 70% of transport groups of pigs. Trailers utilized natural ventilation, and boarding was applied to each trailer as specified by McGlone [8].

The first experiment (January to February 2011; −13 °C to 20 °C) consisted of 2 treatment groups. Treatment 1: 6 bales/trailer (n = 103; 17,100 pigs). Treatment 2: 12 bales/trailer (n = 69; 11,403 pigs). Most (74%) observations were at air temperatures <10 °C. Data collection was done at 2 plants (1 in Iowa, 1 in Missouri) and 32 finishing sites.

The second experiment (March and May 2011, −2 °C to 21 °C) consisted of 3 treatment groups. Treatment 1: 3 bales/trailer (n = 105; 17,745 pigs), Treatment 2: 6 bales/trailer (n = 122; 21,080 pigs), and Treatment 3: 12 bales/trailer (n = 38; 6,075 pigs). Most (69%) observations occurred at air temperatures between 5 °C and 20 °C. Data collection occurred at 1 plant located in Iowa and at 37 finishing sites.

### 2.3. Trailer Characteristics at the Finishing Site and Processing Plant

Bedding levels applied to each trailer, number of loads of pigs (one load is considered a group of pigs loaded and subsequently unloaded from the trailer after transport) that had been previously transported on that bedding, and loading percentage on the trailer was collected at the finishing site and processing plant.

Data on the following aspects of transport was collected both at the finishing site and at the processing plant:

- Bedding level applied to the trailer;
- Number of loads previously transported on the bedding currently in the trailer; and,
- Loading percentage, or percent of intended load that was actually loaded onto the trailer.

### 2.4. Temperature and Relative Humidity Inside the Trailers

Sensors (Extech model RHT10, Extech Instruments, Nashua, NH and HOBO Model H08-003-02, Onset Computer Corp., Bourne, MA) collected temperature and relative humidity every 5 min during loading, waiting time at the farm, transit, waiting time at the plant, and unloading. Sensors were installed inside the trailers in 4 locations. All sensors were placed approximately 1 m above the trailer floor, which allowed data to be collected near the pigs, but were out of reach of the pigs. Two sensors were placed on each deck of the trailer, 1 in the frontmost compartment and 1 in the rearmost compartment of each deck. Sensors were placed opposite of the other sensor on the same deck. Additionally, sensors were placed opposite of the corresponding sensor on the other deck, e.g., if the sensor on the top deck in the frontmost compartment was placed on the left side, then the sensor on the bottom deck in the frontmost compartment would be placed on the right side [9].

Air temperature and relative humidity (RH) were recorded as a single measure at the processing plant upon arrival of each trailer using the Kestrel sensors (Kestrel 4500 Pocket Weather Tracker and Extech model RHT10, Extech Instruments, Nashua, NH). Air temperature and RH are available for 70% of the trailers.

### 2.5. Transport Losses over the Two Experiments

Total numbers of DOA, NA, and total D&D (including dead in pen) pigs for each load were recorded by auditors and matched with plant records. The NA counts included both injured and non-injured pigs as plant protocol did not differentiate between NA and NAI.

### 2.6. Pig Surface Temperature

Skin surface temperatures were taken with a dual laser infrared laser thermometer (Extech model # 42570, Extech Instruments, Nashua, NH, USA) laterally near the midline of randomly selected animals at loading and unloading. As surface temperatures were relatively uniform at farm site (loading), surface temperature data at the plant will be used. Ten pigs were randomly selected from each load, 5 of first 50 unloaded and 5 of last 50 unloaded. Because the trailer was the experimental unit, data from the 10 pigs sampled for surface temperature at the plant were averaged to get a single value per trailer.

### 2.7. Bedding Moisture

Bedding samples were collected for moisture percentage analysis after pigs had been unloaded. Researchers collected 1 sample of at least 100 g of bedding from the center of each deck, ensuring that the entire thickness of the bedding was represented. Samples were stored in resealable zipper storage bags, which in turn were sealed into larger resealable zipper storage bags, so as to prevent moisture loss. The samples were not frozen until the end of the week due to lack of freezer space at the plant; therefore, the time from collection to freezing varied from 1 to 5 days. Samples were stored at −80 °C until analyzed. Moisture percentage was estimated by drying 3 to 4 g of bedding in an aluminum cup at 100 °C for 24 h and calculating the percentage loss of moisture as shown [10]:

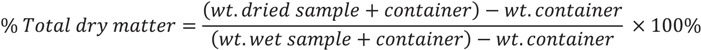

% *total moisture* = 100 − % *total dry matter*

Samples were analyzed in duplicate. If the coefficient of variation (CV) between the samples was greater than 10%, samples were analyzed again in duplicate until the CV was less than 10%.

### 2.8. Statistical Analyses

The statistical model included the effects of bedding level, outside air temperature (in 5 °C bins), and the interaction of bedding level and outside air temperature. The experimental unit was the trailer containing all pigs (average of 166.9 ± 0.63 pigs/trailer) in all studies that examined bedding and air temperature effects. The experimental unit was also the trailer, with a sample of 10 pigs from a given trailer for surface temperature assessment data set. All data were analyzed using SAS 9.2 General Linear Models procedure (SAS, 2010 SAS Inst., Inc., Cary, NC, USA). When the treatment P-values were less than 0.10, a T-test (unprotected if P = 0.10 to 0.05) was performed using the Predicted Difference test within the Least Squares Means procedure of SAS. Regression analyses were calculated to determine linear and quadratic effects for air temperature in relation to DOA, NA, D&D, and pig skin surface temperatures at the plant.

## 3. Results and Discussion

### 3.1. Temperature and relative humidity inside the trailers

Averages and ranges of temperatures and RH inside the trailers during transport for all experiments are presented in Table 1.

**Table 1:** Average and range of temperatures and relative humidity (RH) inside trailers during Experiment 1 (cold) and Experiment 2 (mild) during transportation of finishing pigs.

Experiment	Number of trailers	Temperature [°C] ^2^			RH [%] ^2^		
		Average	Minimum	Maximum	Average	Minimum	Maximum
1 ^1^	172	7.8 ± 0.10	−6.80	26.60	69.2 ± 0.25	23.60	98.80
2 ^1^	265	12.1 ± 0.09	−3.70	33.20	60.9 ± 0.25	19.40	100.00

^1^ Experiment 1 was conducted in January and February 2011. Experiment 2 was conducted in March and May 2011. ^2^ Temperature and RH data were collected at 5 minute intervals via sensors placed throughout the trailer.

### 3.2. Transport Losses over the Three Experiments

#### 3.2.1. Experiment 1—Cold Weather

A total of 166.0 ± 1.98 pigs were transported in trailers with six bales of bedding and 165.36 ± 2.43 pigs in trailers with 12 bales of bedding (*P* = 0.81). There were no observed main effects for bedding level on the trailer, outside air temperature, or the interaction for transport losses in the finishing pig (*P* > 0.13). Table 2 shows the effect of bedding level on transport losses in cold weather.

**Figure 1:**
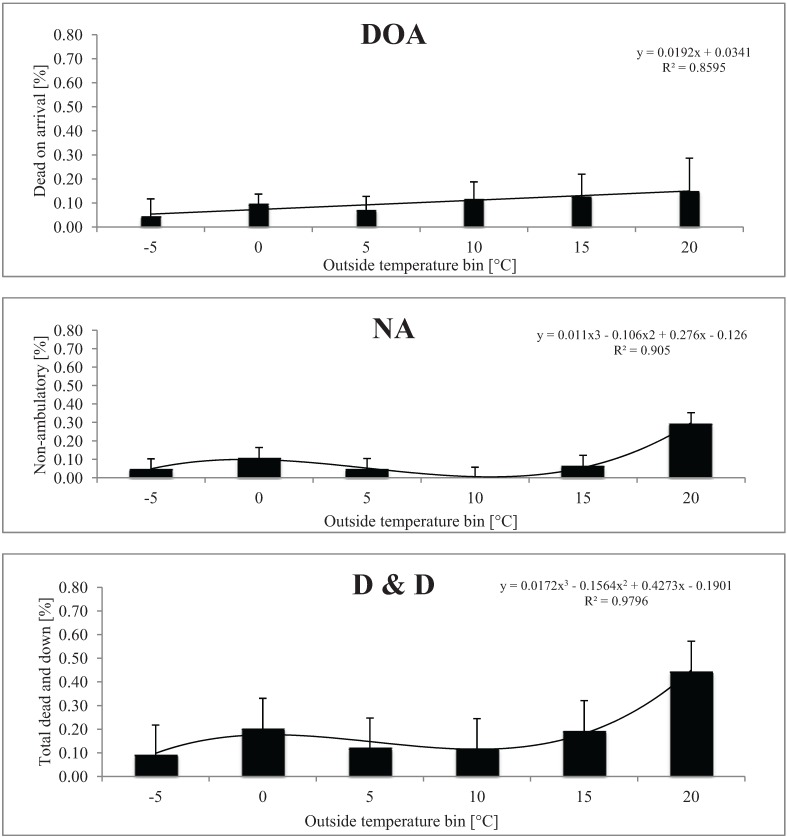
Least square means of dead on arrival (DOA), non-ambulatory (NA), and total dead and down (D&D) pigs, respectively, in relation to outside temperature bins in cold weather. Total number of loads for each temperature bin is given in parentheses: −5 °C (14), 0 °C (44), 5 °C (24), 10 °C (15), 15 °C (9), and 20 °C (4). A total of 110 trailers each carrying an average of 164.28 pigs were used for this study.

The percentage of DOA, NA, and D&D pigs in relation to outside temperature bins in cold weather is presented in Figure 1.

**Table 2:** Effect of bedding level on the percentage of dead on arrival (DOA), non-ambulatory(NA), and total dead and down (D&D) pigs during Experiment 1 (cold weather, air temperature −13 °C to 20 °C).

Condition upon arrival [%]	Bedding level [bales ^1^/trailer]		SEp	*P*-values		
	6	12		Bedding	Temperature	Bedding × Temperature
No. of trailers	103	69				
No. of pigs	17,100	11,403				
DOA	0.06	0.12	0.03	0.13	0.95	0.56
NA	0.05	0.07	0.03	0.73	0.53	0.54
D&D	0.11	0.18	0.04	0.29	0.73	0.47

^1^ One bale = 22.7 kg, 0.2 m^3^.

#### 3.2.2. Experiment 2—Mild Weather

There was a difference in the number of pigs loaded with three (169.0 ± 1.98 pigs), six (172.8 ± 1.83 pigs), and 12 (159.9 ± 3.29 pigs) bales of bedding (*P* < 0.01). There was no observed main effect for bedding or for the bedding by temperature interaction on transport losses in the finishing pig (Table 3) (*P* > 0.05). There was no temperature effect on DOA (P = 0.86); however temperature did affect NA (*P* < 0.01), and tended to affect D&D (*P* = 0.07). Table 3 shows the effect of bedding level on types of losses in mild weather.

**Table 3:** Effect of bedding level on percentage of dead on arrival (DOA), non-ambulatory (NA), and total dead and down (D&D) pigs during Experiment 2 (mild weather, air temperature −2 °C to 21 °C).

Condition upon arrival [%]	Bedding level [bales ^1^/trailer]			SEp	*P*-values		
	3	6	12		Bedding	Temperature	Bedding × Temperature
No. of trailers	105	122	38				
No. of pigs	17,745	21,080	6,075				
DOA	0.11	0.12	0.03	0.03	0.21	0.86	0.30
NA	0.09	0.10	0.13	0.03	0.84	0.01	0.19
D&D	0.20	0.22	0.09	0.05	0.34	0.07	0.17

^1^ One bale = 22.7 kg, 0.2 m^3^

**Figure 2:**
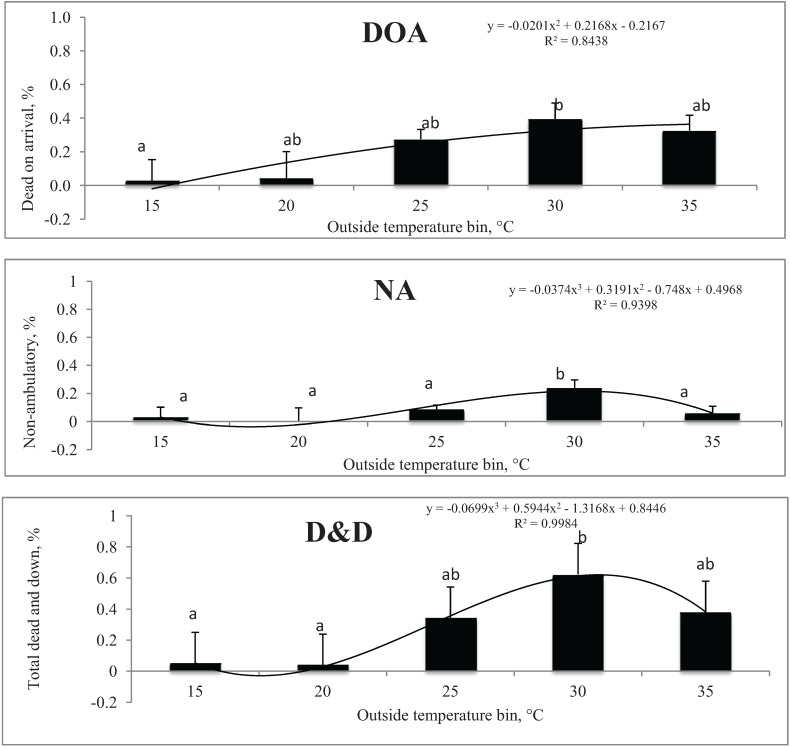
Least square means of Dead on arrival (%), Non-ambulatory (%), Total dead and down (%), respectively, in relation to outside temperature bins in mild weather. Total number of loads for each temperature bins represented in parenthesis: 0(6), 5(23), 10(29), 15(47), 20(27), 25(2). 134 trailers with 32,962 (average = 171.67) pigs were used for this study.

Figure 2 represents percentages of DOA, NA, and D&D pigs in relation to outside temperature bins in mild weather.

### 3.3. Pig Surface Temperature at the Plant

In cold weather there was no difference for surface temperatures at the plants for pigs transported with six *vs*. 12 bales (23.8 ± 0.85 °C *vs*. 23.98 ± 0.98 °C) (*P* = 0.96).

In mild weather, skin surface temperature of pigs transported with three or six bales of bedding was not different and skin surface temperature of pigs transported with 12 bales of bedding was lower than that of pigs transported with three or six bales (*P* < 0.05). Surface temperatures differed at the plant (28.9 ± 0.50 °C for three bales, 29.2 ± 0.49 °C for six bales, and 25.9 ± 0.84 °C for 12 bales (*P* < 0.01)) but were not different at the finishing site (*P* > 0.05).

### 3.4. Overall Effect of Air Temperature

Pig surface temperature at the plant responded to outside air temperature with approximately a 0.37 °C increase per 1.0 °C increase in outside air temperature. The DOA, NA, and D&D percentages increased as outside temperature increased at the plant (*P* < 0.01, 0.05, and < 0.01, respectively). A sharp rise in D&D rates was observed when pig surface temperature exceeded 32 °C.

**Figure 3:**
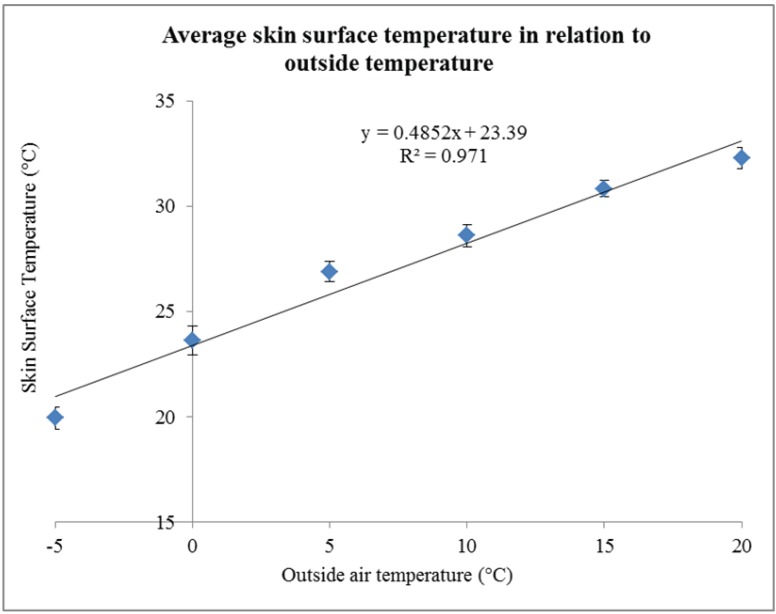
Skin surface temperature of pigs in relation to outside air temperature when unloaded at the processing plant. Total number of loads for each temperature bin is given in parentheses: −5 °C (14), 0 °C (14), 5 °C (31) 10 °C (25), 15 °C (70), 20 °C (39), and 25 °C (86). A total of 335 loads with 56,106 pigs were used for this analysis.

When skin surface temperature at the plant was plotted against outside air temperatures for all temperature bins (−5 °C to 20 °C, in 5°C bins), skin surface temperature increased linearly with increasing outside air temperature (*P* < 0.01). The regression line linear fit had an r^2^ of 0.95, which indicates a strong relationship. In addition, above 30 °C, D&D increased linearly with increasing skin surface temperature after transportation (r^2^ = 0.81), indicating that skin surface temperature can be used as an indicator of D&D following transportation. This method does not apply to losses occurring on the trailer (DOA, specifically), but may be beneficial in determining if cooling interventions are necessary when pigs arrive at their destination. Figure 3 shows the linear relationship between skin surface temperature and outside air temperature at the processing plant.

### 3.5. Bedding Moisture

Bedding samples were not collected in January, and 42 samples were available for moisture analysis in February. Of these samples, average moisture content in the bedding after pigs were unloaded was 60.4 ± 1.86% (minimum 35.8%, maximum 74.4%). Of 213 samples collected and analyzed in March and May, average moisture content in the bedding collected at the plant after unloading was 58.2 ± 1.07% (minimum 25.6%, maximum 80.8%). Moisture content of the bedding did not have a significant effect on losses in cold or mild temperatures.

## 4. Conclusions

Ethical and economical transport of finishing pigs is an important aspect of swine production and welfare. Annual transportation losses in the U.S. were estimated at approximately $46 million in 2006 [2]. Transportation is a known stressor for swine that has been previously assessed measuring metabolic products such as cortisol, and by determining decreases in carcass yield and the percentage of DOA, NAI, and NANI pigs [6,11,12]. Factors like stocking density, travel distance, total time to transport, feed withdrawal, handling at loading and unloading, season, trailer type, and microenvironment have been identified as factors associated with in-transit losses [13,14,15].

The results of this study showed that in cold weather, bedding level neither improved nor worsened the percentages of DOA, NA, and D&D. Based on this finding it would be advisable to provide six bales of bedding in cold weather, as there is no proven benefit of providing more. However, there is no data on rates of losses with less than six bales of bedding; therefore, more research is needed on fewer than six bales of bedding to make an accurate recommendation.

There was no effect of bedding level in mild weather. This study showed that there is no advantage of providing more than three bales of bedding. As in cold weather, there is no available data for rates of losses when animals are transported with less than three bales, so more research is needed to provide an accurate recommendation.

The findings of the present study agree with those of Fitzgerald [16], in that the percentage of total losses per trailer was greater during winter compared to mild weather. Also, losses during winter were mainly a result of fatigued pigs, possibly attributed to shivering. Past research correlates in-transit losses with stocking density, season, trailer design, and ventilation [17], but no studies have measured in-transit losses associated with the bedding level used. Results obtained from this study showed that higher levels of bedding did not result in fewer losses in terms of DOA, NA, and D&D during winter as opposed to the bedding levels recommended by the TQA handbook. Similarly, no benefit was observed from using higher levels of bedding during mild weather. The present study showed that skin surface temperature increased linearly by 0.37 °C for every 1.0 °C increase in outside air temperature during unloading at the processing plant. In a preliminary study, skin surface temperature was shown to increase by 0.19 °C for every 1.0°C rise in outside air temperature after being transported for 14 h [18]. Chung *et al.* [19] determined that skin surface temperature could be used to predict rectal temperature after piglets are transported. The results of their study suggested that rectal temperature increases by 0.30 °C for every 1 °C increase in abdominal surface temperature. In addition, D&D rate during the summer increased linearly with skin surface temperature above 30 °C, suggesting that this relationship may be useful as a means of predicting D&D rates during summer transportation.

When bedding samples were assessed for moisture content after transportation, it was found that moisture levels were approximately 2-fold greater after the second load of pigs when compared to the first. Unused bedding contained approximately 5% moisture. After the first load moisture content increased to 26%. After two loads of pigs had been transported on the same bedding, moisture content was nearly 51%. This moisture in the bedding may contribute to transport losses in winter, where the dampness combined with cold air may cause the pigs to shiver more, supporting the knowledge that winter losses are primarily a result of fatigued pigs. Reuse of bedding occurs due to the costs of washing out and applying new bedding to a trailer after each transport. Kephart *et al.* [20] estimates the annual cost of washing out and rebidding to be between 22 and 135 million dollars.

Overall, excessive use of bedding should be avoided. It has no direct benefit to the animals, and may even be harmful. The authors of this paper determined that overuse of bedding results in approximately $4 million in extra expenses annually. This represents a large economic impact to the transport industry. The current study needs to be expanded upon in order to make accurate recommendations for transport during each season and in different climate ranges.
